# Pathogenesis of Malaria in Tissues and Blood

**DOI:** 10.4084/MJHID.2012.061

**Published:** 2012-10-04

**Authors:** Beatrice Autino, Yolanda Corbett, Francesco Castelli, Donatella Taramelli

**Affiliations:** 1 University Division of Infectious and Tropical Diseases, University of Brescia and Spedali Civili General Hospital, Brescia (Italy), Piazza Spedali Civili, 1 - 25123–Brescia, Italy; 2 Dipartimento di Scienze Farmacologiche e Biomolecolari, Università di Milano, Via Pascal 36, 20133 Milano Italy; 3 Chair of Infectious Diseases, University of Brescia, Italy

## Abstract

The clinical manifestations of severe malaria are several and occur in different anatomical sites. Both parasite- and host-related factors contribute to the pathogenicity of the severe forms of the disease. Cytoadherence of infected red blood cells to the vascular endothelium of different organs and rosetting are unique features of malaria parasites which are likely to contribute to the vascular damage and the consequent excessive inflammatory/immune response of the host. In addition to cerebral malaria or severe anaemia, which are quite common manifestation of severe malaria, clinical evidences of thrombocytopenia, acute respiratory distress syndrome (ARDS), liver and kidney disease, are reported. In primigravidae from endemic areas, life threatening placental malaria may also be present.

In the following pages, some of the pathogenetic aspects will be briefly reviewed and then data on selected and less frequent manifestation of severe malaria, such as liver or renal failure or ARDS will be discussed.

## Introduction

Malaria is a life-threatening disease which in 2010 killed more than 600,000 individuals, mainly children under 5 years of age, and pregnant women.^1^ All the clinical symptoms of malaria are the consequence of infection of human erythrocyte by merozoites. Most of the fatal cases, which predominantly occur in *P. falciparum* infections, are due to severe anaemia or cerebral malaria, but different clinical manifestations also exist and vary in severity and outcome, depending on the parasite species, the organ involved and the access to care.

*P. falciparum* differs from other human malarial species in that infected red blood cells (IRBC) do not remain in the circulation for the entire life cycle. After 24–32 hours, when young parasites mature from the ring to the trophozoite stage, IRBC adhere to endothelial cells in the microcirculation of various organs. This phenomenon, termed “sequestration”, is believed to occur mainly to avoid splenic removal of IRBC. Sequestration causes microcirculatory obstruction, impaired tissue perfusion and inflammatory cells activation and it is linked to the severity of the disease.

At schizonts rupture, from 4 up to 36 daughter merozoites, depending on the Plasmodium species, are released into the circulation and invade fresh RBC to perpetuate the asexual life cycle. At the same time, a large amount of toxins and parasite products are also released and cause the activation of the innate immunity, the release of inflammatory mediators and the symptoms associated with the malaria attack, such as fever.

A combination of mechanical circulatory stress due to sequestration and excess inflammatory response contribute to the most severe manifestations of malaria including, but not limited to, cerebral malaria or anaemia. The present review will summarise some of the pathogenetic aspects of severe malaria and then it will report on selected and less frequent manifestation, such as renal failure or acute respiratory distress syndrome (ARDS).

## Pathogenetic Characteristics of Severe Malaria

### Cytoadherence

Cytoadherence, the ability of parasites to adhere to the vascular endothelium, was recognized as early as 1892 by Marchiafava and Bignami.^2^ Mature forms of parasites (asexual stage and gametocytes) can adhere to the vascular endothelium of several organs (lung, heart, brain, lung, liver, and kidney), the subcutaneous adipose tissues and the placenta. This feature of the disease *in vivo* has been related exclusively to *P. falciparum*.^3^,^4^ However, sequestration *in vitro* to some endothelial cell lines and placental cryosections has also been seen in reticulocytes infected with *P. vivax*.^5^

Parasite sequestration is thought to be the pathological base of the severe manifestation of malaria, including cerebral malaria.^6^ It causes blood flow impairment leading to local hypoxia. It enhances parasite replication and the sticking of IRBC to non-infected red blood cells (rosetting, see below). Moreover, when parasite sequester, the effects of parasite toxins are more localized and also the stimulation of the host immune response, which may cause a focused production of inflammatory mediators and tissue damage. As a consequence, both RBC and IRBC become more rigid and less deformable.^7^

Sequestration is mostly mediated by mature parasite forms, approximately 20 hours after RBC invasion. The parasites produce new proteins that are exported to the IRBC surface and increase the adhesiveness of IRBC to the endothelium. During their 48-hour life cycle, the parasites can remain sequestered for 24 hours in the deep microvasculature. In this manner, they evade clearance by the spleen, and make the diagnosis more difficult since they are not seen in the peripheral blood.

Sequestration of *P. falciparum* has been attributed to different class of molecules of parasite origin and ligands present on the human endothelium. Among those, the *P. falciparum* histidine-rich protein (PfHRP) and the erythrocyte membrane protein 1 (PfEMP1), have received significant attention. PfHRP is related to the establishment of knobs, symmetric membrane arrangements which appear on the surface of infected RBC, while PfEMP1, a multimeric protein encoded by the *var* (variant) gene^3^,^4^ protrudes from the knobs and plays a major role in sequestration and thus virulence (see BOX 1). To adhere to the endothelium, the parasites first adhere, roll and then become firmly attached to the endothelium adhesion molecules. Among these molecules, ICAM-1, a major sequestration receptor and involved in cerebral sequestration serves as a rolling receptor. On the other hand, CD36 gives stationary and stable adherence under flow.^8^–^10^

Box 1 **PfEMP1, a molecule for antigenic variation, adhesion and immune modulation**

- *P. falciparum* erythrocyte membrane protein 1 (PfEMP1) is a multimeric protein encoded by a family of roughly 60 variant (*var*) genes.^4^
- Each individual parasite expresses a single *var* gene at a time, maintaining the remaining *var* genes in a transcriptionally silent state. Epigenetic factors control the exchanges in the expression of the *var* gene generating antigenic variation of the IRBCs surface.^4^
- PfEMP1 is localized in the Maurer’s clefts, from where it traffics to the erythrocyte membrane, and on the IRBC surface. Together with knob-associated histidine-rich proteins (HRPII), PfEMP1 forms protrusions, known as knobs.^133^
- PfEMP1 is crucial for malaria virulence and pathogenesis.^134^
- PfEMP1 mediates the adhesion to the endothelium, so parasites avoid destruction by the immune system and the spleen, and also their detection in the peripheral blood. The multiple adhesion domains are localized in the extracellular portion of PfEMP1 which is recognized by host receptors. There are approximately 11 receptors involved in adhesion of PfEMP1 to the endothelium. CD36, ICAM-1, chondroitin sulphate A (CSA) and trombospondin are particularly important for sequestration; whereas CR1, the complement receptor is involved in rosetting.^15^,^135^

Sequestration is also seen during gestational malaria, when parasites adhere to the placenta. PfEMP1 is again the main adhesion receptor which adheres to the trophoblastic villous endothelium mainly through chondroitin-4-sulfate (CSA) and other sugars such as glycosaminoglycans and possibly hyaluronic acid (HA). As discussed later, malaria in pregnancy can be severe for mothers and induce fetal death especially during the first pregnancy, when women usually lack sufficient immunity against CSA-binding parasites.^11^–^14^

### Rosetting

Rosetting is one of the forms of cytoadherence of late stages IRBC to non-parasitized red blood cells and/or platelets.^15^ The IRBC ligand involved in rosette formation is PfEMP1, and three are the receptors associated with rosetting: complement receptor 1 (CR1), heparan sulfate (HS), and the ABO blood group.^15^,^16^ PfEMP1 has been shown to bind to CR1, specifically at the C3b-binding site. The lectin-like DBL-domain of PfEMP1 can make strong adhesion with carbohydrate structures particularly A blood group antigen, favoring rosettes formation.^17^ For this reason, non-O blood groups are considered significant risk factors for life-threatening malaria, through the mechanism of enhanced rosette formation.^18^,^19^

*P. falciparum, P. vivax*, and *P. ovale* are all able to form rosettes,^20^,^21^ but only those caused by *P. falciparum* have been associated with severe malaria, and especially in African children they may enhance microcirculatory obstruction.^22^ Rosetting has been related with parasite multiplication rate in a model of *Saimiri sciureus*.^23^ More recently, it has been shown that 4-HNE, a biomembrane lipid peroxidation product driven by haem iron of the malarial pigment can be transferred from IRBC to normal RBC in rosettes favouring their removal by macrophages.^24^ This could partly explain the rapid loss of normal non parasitized RBC in severe malaria anaemia.

### Innate Immune Response

The immune response to the parasite is complex and not completely understood, and it is essentially both species and stage specific.^25^ The activation of components of the innate immune system is crucial to control parasite replication, contributing to the subsequent elimination and resolution of the infection.^26^ Neutrophils, monocytes/macrophages, dendritic cells, natural killer (NK) cells, NKT cells, and gamma T cells are all the cells of the innate immune system in charge of controlling the early progression of the disease through phagocytosis and/or production of inflammatory mediators. Much of the symptoms of malaria attacks such as fever, nausea, headaches, and others are the consequences of the inflammatory response orchestrated by the cells of the innate immune system, stimulated by parasites or their products at the rupture of the late stage infected erythrocytes.^25^,^27^ An imbalance between the production of pro- or anti-inflammatory cytokines, such as TNF-α, IL6, IL1β, or IL-10 or mediators, like nitric oxide, may contribute to the pathogenesis of the severe form of the disease. Elevated levels of TNF-α are found in the serum of severe patients and have been correlated with cerebral malaria (CM).^28^ *In vitro*, it has been found that TNF-α enhances the expression of ICAM-1,^29^ which is also augmented in brain vessels of CM patients.^9^ Evolutionarily conserved receptors, termed “pathogen recognition receptors” (PRRs), are present on the cells of native immunity and trigger the response upon the recognition of specific parasite molecules called “pathogen associated molecular patterns” (PAMPs).^30^ Malaria PAMPs include the protein anchor, glycosyl-phosphatidyl inositol (GPI), or hemozoin (malaria pigment). PRRs related to malaria are the membrane bound Toll-like receptors (TLRs), the cytosolic receptors (such as NALP3, inflammasomes), and soluble receptors such as MBL.^31^–^34^ For example, GPI released by IRBC at shizonts rupture stimulates the production of pro-infiammatory TNF-α by macrophages via the recognition of TLR2 and partly of TLR4 in a MyD88 dependent way.^31^

### Specific Immune Response

Malaria is an important cause of morbidity, but not everyone infected with the malaria parasite becomes seriously ill or dies. In areas of stable endemicity, repeated exposure to the parasite leads to the acquisition of specific immunity, which restricts serious problems to young children; malaria in older subjects causes a relatively mild febrile illness. However, individuals with no previous experience of malaria become ill on their first exposure to the plasmodium parasite. They develop a febrile illness which may become severe and in a proportion of cases may lead to death.^35^,^36^

Immunity to malaria is provided by innate mechanisms, as we summarized above, and subsequently by the development of acquired immunity. Following repeated infections, people living in malaria endemic areas gradually acquire mechanisms, which helps limiting the inflammatory response to the parasite that causes the acute febrile symptoms. Sterilizing immunity is never achieved. Parasites have evolved to maintain a balanced relationship with their human hosts. In this sense they can partially escape from the host effector mechanisms, while hosts are able to develop partial immunity against the parasite. This type of immunity requires repeated infections, takes years to develop and usually lasts shortly. Natural acquired immunity is called premunition since low parasite burden often persists in the presence of circulating antibodies to the various stages, in the absence of clinical disease. In children whithout circulating antibodies to the parasite, premunition is lower. The poor and slowly developing immune response to malaria is partly due to parasite immune evasion strategies: antigenic polymorphism, shedding of parts of parasite proteins, cross-reactive antigen epitopes of developmental stages, prolonged exposure to endemic malaria and limited immunogenicity of antigens. In stable endemic areas, a heavy burden of morbidity and mortality falls on young children. Children born to immune mothers appear to be relatively immune to malaria for a period.^26^,^37^ This is conferred by the prenatal or postnatal transfer of protective antibodies from mother to child. The acquired immunity is mediated by specific antibodies to several conserved and polymorphic proteins, and to the highly variable protein PfEMP1 expressed by the parasite at the trophozoites and schizonts stages and exported on the surface of infected erythrocytes.^38^

## Specific Complications of Severe Malaria Infection

### Anaemia

Anaemia is one of the most common causes of morbidity and mortality in malaria infection particularly in pregnant women and in children.^39^ Pathogenesis of malarial anaemia has been intensively studied, even if it is not completely understood; hereby we summarise some of the pathogenetic aspects. Malarial anaemia could be acute or chronic; in holo-endemic areas chronic malarial anaemia is more common. Acute malarial anaemia could occur after massive erythrocytes lysis due to elevate parasitemia or to drug-induced or immune haemolysis.^40^

The potential mechanisms contributing to malarial anaemia can be divided into two categories: increased destruction of parasitized and un-parasitized erythrocytes (immune-mediated lysis, phagocytosis, splenic sequestration) and decrease of RBC production (dyserythropoiesis and bone marrow suppression, inadequate reticulocyte production, effects of inflammatory cytokines, effects of parasite factors). Co-infection with bacteremia, HIV-1 and hookworm, malnutrition and repeated malarial infections in holo-endemic countries may also contribute to decrease haemoglobin levels.^39^,^41^–^43^

Parasitized red cells rupture caused by plasmodium cycles and clearance of deformed parasitized and un-parasitized erythrocytes are the principal causes of malarial anaemia. Phospholipid asymmetry, membrane rigidity and reduced deformability are the mechanisms involved in the premature removal of un-parasitized red cell.^7^,^41^,^44^ Phagocytosis and complement activation are the principal mechanisms of non-specific immune mediated clearance of erythrocytes in malaria infection.^45^ Moreover, uninfected red cells membrane proteins may be altered by reactive oxygen species (ROS) and other factors, becoming target for auto-antibodies.^4^ It has been suggested that the spleen may play a double role in malaria infection: on the one hand it could contribute to severe anaemia, by excess removal of IRBC and uninfected RBC; on the other hand it may protect from severe cerebral malaria.^46^

Dyserythropoiesis plays an important role in the pathogenesis of anaemia; examination of bone marrow from children with severe anaemia showed hypercellularity, mild to normal erythroid hyperplasia and abnormal features of late erythroid progenitors. Hemozoin and its phagocytosis by bone marrow macrophages has been proposed to cause dyserythropoiesis either through direct accumulation in the bone marrow and generation of reactive toxic species or activation of the innate immune response.^42^

The immune response is central in the pathogenesis of malarial anaemia; parasitized red cells, hemozoin and malarial antigens activate monocyte and lymphocyte response. Pro-inflammatory and anti-inflammatory mediators, including TNF-α, IFN-γ, IL-23 and IL-1, chemokines and growth factor are produced and contribute to anaemia. On the contrary, IL-12 and IL-10 seems to be protective cytokines since low levels are found in severe malarial anaemia.^40^, ^43^ Macrophage migration inhibiting factor (MIF) is associated with severe anaemia and bone marrow suppression.^47^ Nitric oxide is an inhibitor of erythropoiesis.^40^,^48^

Erythropoietin (EPO) levels are increased during malaria anaemia, but erythroid progenitors response is not adequate, particularly during chronic malaria infection, resulting in low reticulocytosis.^43^ It has been shown in a rodent model that exogenous EPO could stimulate splenic erythroblasts. However, their maturation is impaired due to altered iron metabolism and haemoglobin production.^49^ Ineffective erythropoiesis, erythrophagocytosis and iron delocalisation are the most important causes of reticulopenia.

Pro-inflammatory cytokines play an important role also in iron delocalisation pathway of malarial anaemia. TNFα induces re-localisation of ferroportin, an important protein abundant in the reticulo-endothelial system that mediates macrophage iron release and intestinal iron absorption. Relocalisation of ferroportin induces decrease of iron absorption and release from macrophage cells;^45^ hepcidin, a protein released during chronic disease, also induces reduction of ferroportin and its levels are increased during severe malaria anaemia.^50^

STAT6, a member of signal transducer and activator of transcription family proteins, seems also to be involved in malarial anaemia through the activation of the regulatory cytokines, in particular IL-4 and IFN-γ, resulting in erythropoietic suppression during blood stage malaria.^51^,^52^ A recent review found an interesting correlation between malarial anaemia and micronutrient malnutrition: vitamin A and E, iron, zinc, riboflavin and folate deficiency may play a role in worsening the anaemia mediated by alteration of immunity, dyserythropoiesis and iron metabolism 39. It has also been suggested that high catecholamine concentration could alter the functions of the erythrocyte membrane, providing an additional erythrocyte clearance mechanism.^53^

### Thrombocytopenia

Thrombocytopenia is very common in malaria, usually during the early stage of P. *falciparum* and *P. vivax* infections. Incidence is high both in children and in adults.^54^–^56^ Thrombocytopenia in pregnant women is not well documented, but a recent study performed in Thailand showed that platelet counts were lower in pregnant than in non-pregnant women.^57^ The pathogenesis of malaria thrombocytopenia is complex and may be related to coagulation disturbances, splenomegaly and platelet destruction by macrophages, bone marrow alterations, antibody-mediated platelet destruction, oxidative stress and platelets aggregation. These processes are well described in a recent review.^58^ A study performed in Indonesia showed that patients with *P. falciparum* and *P. vivax* malaria had lower platelet count, higher von Willebrand factor (VWF) concentration, lower ADAMTS13 activity and ADAMTS13 antigen concentrations.^59^ Higher VWF seems to correlate with platelet binding, leading to thrombocytopenia. In contrast, another study demonstrated that sGP1b, the external domain of platelet receptor for VWF, increased early in the blood of malaria patients thus preventing excessive platelet adhesion.^60^ Despite thrombocytopenia is very common, hemorrhagic events are rare and usually are associated with severe thrombocytopenia or disseminated intravascular coagulation (DIC).^61^–^63^ Further study are needed to understand the relationship between malaria, platelet counts and hemorrhagic events.

### ARDS

Deep breathing, respiratory distress and pulmonary oedema are some of the clinical feature defining severe malaria according with WHO classification.^64^ In adults and pregnant women, rather than children, acute lung injury (ALI) and acute respiratory distress syndrome (ARDS) are the most common clinical presentation burdened by an elevated mortality rate as shown in a recent study performed in India where severe *P. falciparum* malaria mortality rate was 35,4% and mortality was principally due to shock, acute renal failure, seizure and ARDS.^65^ Despite ARDS is mostly observed as a complication of *P. falciparum* infection, case reports due to *P. vivax*,^66^–^69^ *P. ovale*^70^ and *P. knowlesi*^71^,^72^ were also published. Malaria associated ARDS due to *P. vivax* caused three maternal deaths in a cohort of 221 patient in India during one year of observation.^66^ ARDS can occur before or after specific treatment.^67^ *P. knowlesi*, the fifth plasmodium species infecting humans, mostly in Southeast Asia, may cause life threatening diseases, as well.^73^ A prospective study conducted in Malaysia demonstrated that the most frequent complication of *P. knowlesi* infection was respiratory distress.^72^ Malaria is presently considered one of the most common risk factor of ARDS and acute lung injury (ALI) in the tropics^74^ and the second cause of ARDS after sepsis.^75^

Little is known about the pathogenesis of malaria associated ALI/ARDS. Inflammatory mediators and increased endothelial permeability may play an important role, while parasite sequestration may take a minor role. Pulmonary manifestations of uncomplicated malaria were analysed by Anstey *et al*. showing that patients frequently have subclinical impairment of lung function, such as small airways obstruction, impaired alveolar ventilation, reduced gas exchange and increased pulmonary phagocytic activity.^76^ All these features underline the activation of inflammatory pathways and could explain part of the pathogenetic mechanisms of respiratory distress occurring during severe malaria. Studies with rodent malaria models were performed to understand the pathogenetic mechanisms of ALI/ARDS. Epiphanio *et al*. in DBA/2 mice infected by *P. berghei* ANKA 77 observed that 60% of mice had dyspnea, airways obstruction and hypoxemia; pleural effusion, pulmonary hemorrhagies and oedema were also found as pathological findings, demonstrating the role of inflammation in ALI development. Moreover, high levels of vascular endothelial growth factor (VEGF) were found in mice with ALI, supporting the importance of increased vascular permeability in malaria respiratory failure. Another study^78^ which utilized DBA/2 mice infected with *P. berghei* K173, showed that proteins and inflammatory cells mainly CD4+ and CD8+ lymphocytes, neutrophils and monocytes accumulate in the lungs of infected mice. These results were confirmed by P.E. Van den Steen *et al*.,^79^ who also measured the cytokines and chemokines associated with ARDS, showing an increased expression in the lungs of TNF-α, IL-10, IFN γ, CXCL10 and CXCL11, as well as monocyte and neutrophil chemo-attractant chemokines (CCL2, KC). IRBC were are also observed in the lung vessels, but at a lower extent compared with the massive sequestration in the brain.^80^ Infected mice lungs had an increased water content, demonstrating the development of oedema;^78^,^79^ this could be related to the decreased expression of epithelial sodium channel, ENaC, due to hypoxia, resulting from malaria associated anaemia, or to TNFα mediated down-regulation.^78^ In a rodent model malaria associated ARDS, dexamethasone inhibited the infiltration of macrophages and CD8 T cells into the lungs, suggesting that adjunct therapy with anti-inflammatory drugs could also be useful in the clinical setting.^79^

Most of the studies with *P. falciparum* are based on *in vitro* models or *post mortem* observations. It was shown i*n vitro* that *P. falciparum* merozoite proteins could increase endothelial permeability, while *P. falciparum* IRBC did not show the same properties suggesting that the effects of the parasite on the pulmonary endothelium are probably mediated by the activity of Src-family kinases.^81^ Morphologic changes were noted in the proteins of the tight junctions and adherent junction in association with increased endothelial permeability and development of pulmonary oedema.^81^ A post mortem study performed in children with cerebral malaria demonstrated that sequestration of *P. falciparum* -IRBC occurs in the lungs, even if to a lesser extent than in the brain, skin or intestine.^80^

Different pathological presentations of ARDS were observed among the different species of human malaria: the greatest severity and frequency of cases are due to *P. falciparum* and could be partially attributed to the sequestration and rosetting of infected RBC in the pulmonary microcirculation;^67^ heavy parasitaemia and WBC agglutinates were associated to ARDS in *P. vivax* malaria.^66^ L. Price *et al*. suggested that the pathogenesis of lung injury by *P. falciparum* include cytokine-induced damage or direct effects of sequestration of parasitized erythrocytes, while ARDS caused by *P. vivax* may be due principally to dysregulation of cytokines production.^69^ Elevated parasitemia in ARDS in *P. knowlesi* infection suggests parasite-specific effects that increase pulmonary capillary permeability,^71^ but hypoxemia and metabolic acidosis may also contribute.^72^

### Liver involvement

According to the World Health Organization liver dysfunction is an uncommon occurrence in malaria, while jaundice is not unusual. The incidence of jaundice and liver dysfunction in *P. falciparum* malaria varied from 5.3% to 62% and from 2.5% to 21%, respectively, in different reports,^82^–^84^ while malarial hepatitis is rare in *P. vivax* infection.^85^ Case-fatality rate in malaria-related hepatic failure is elevated, up to 40%^84^–^86^ when high parasite density is associated with jaundice and liver dysfunction.^87^ Liver is involved in malaria at two stages: during the pre-erythrocytic cycle and the erythrocytic phase. The first step is linked to the binding of the merozoite circumsporozoite protein CSP-A and TRAP protein to the hepatocytes via the heparan sulphate glycosylaminoglycans GAG^88^ and promotes minimal liver damage. In the erythrocytic phase, jaundice is a common remark and it is directly caused by the infection (malarial hepatitis, intravascular hemolysis of parasitized RBC, septicemic hepatitis), or by indirect causes (microangiopathic hemolysis associated with DIC, G6PD-related hemolysis, antimalarial drug induced-hemolysis) or completely unrelated (coexisting acute viral hepatitis, underlying chronic hepatitis).^88^ Intravascular hemolysis of parasitized and non parasitized RBC causes an increase of unconjugated bilirubinemia with mild to moderate jaundice;^89^ conjugated hyperbilirubinemia indicates hepatocyte dysfunction. The pathogenesis of hepatic dysfunction is not completely known; reduction in portal venous flow as a consequence of microocclusion of portal venous branches by parasitized erythrocytes, intrahepatic cholestasis due to reticuloendothelial blockage and hepatic microvilli dysfunction, suppression of bilirubin excretion due to effect of parasitemia or endotoxemia or metabolic acidosis, apoptosis and oxidative stress are all mechanisms involved in hepatic damage.^88^,^90^ Histopathological findings reported in literature are: congestion of hepatocytes, swollen hepatocytes, centrizonal necrosis, Kupffer cell hyperplasia, deposition of brown malarial pigment, portal infiltration with lymphocytes, steatosis, parasitized RBC, cholestasis, spotty and submassive necrosis.^88^–^91^ These evidences demonstrate inflammatory as well as direct plasmodial effects in the damage to hepatocytes.

### Kidney involvement

Kidneys in malaria are involved in two different manners: acute and chronic diseases. Acute renal failure (ARF) is one of the most challenging diseases in tropical countries and malaria plays an important epidemiological role.^92^–^95^ The mortality due to malaria ARF is high.^96^ A study performed in Yemen showed that malaria was the first cause of death in patient with ARF.^97^ Malaria acute renal failure (MARF) is more common in non-immune adults and in older children.^98^ MARF is mostly associated to *P. falciparum* infection and is more frequent in low transmission areas;^98^–^101^ nevertheless MARF could be caused also by *P. vivax*.^100^,^102^ *P. malariae* causes chronic and progressive glomerulopathy, known as quartan malaria nephropathy (QMN); only few cases of MARF caused by *P. malariae* has been published.^103^

Quartan malaria nephropathy (QMN) is frequently seen in African children and it is clinically associated with oedema and hypertension; urine analysis shows often proteinuria and microhematuria.^101^,^104^ Pathogenesis of QMN is linked to subendothelial deposits of immune complexes containing IgG, IgM, C3 and in the 25–33% of cases also malaria antigens.^101^,^104^ It is thought to be the consequence of the activation of TH2 type T lymphocytes. The pathogenesis may also include genetic and acquired factors, such as autoimmunity, co-infection with Epstein-Barr virus and malnutrition.^104^ Immune complexes deposition provokes glomerular damage, resulting in proliferative glomerulonephritis; the pathology, initially focal, becomes diffuse and progressive, reaching sclerosis. Different histopatological patterns revealed by renal biopsy induced some authors to conclude that the association between *P. malariae* infection and renal involvement was only coincidental.^101^ *P. falciparum* acute renal failure is more common in adults; its pathogenesis is complex and it includes mechanical and immunologic factors, volume depletion, hypoxia, hyperparasitemia and other factors.^98^,^101^,^105^ High parasite density was associated to acute renal failure.^101^,^87^ Parasitized erythrocyte sequestration was found in kidneys of adults who died from severe *P. falciparum* malaria;^106^ sequestration of parasitized erythrocytes is lower in the kidney than in the brain.^98^,^106^ A study performed in Mali, showed that rosetting is present in all severe malaria clinical forms, including acute renal failure.^22^ Hemolysis, causing endothelial activation and hemodynamic alteration, can lead to acute tubular necrosis and acute interstitial nephritis.^104^ Hypoperfusion, due to the loss of liquids and to the absence of volume restoration, leads to renal ischemia.^98^ TNFα, reactive oxygen species and inducible nitric oxide also play an important role in determining the haemodynamic alteration.^104^ Mononuclear cell infiltration was reported in glomerular and in peritubular capillaries of adults with ARF, while immune complexes were not reported.^106^ It was hypothesized that the release of malaria antigens activates monocyte cells, to release proinflammatory cytokines and activate TH1 cell mediated response, causing acute interstitial nephritis.^104^ The role of cytokines such us INFγ, IL-1α, IL-6, GM-CSF was studied in murine malaria infection and an association of nephritis with up-regulation of pro-inflammatory and dysregulation of anti-inflammatory cytokines was found.^107^

Rarely, acute renal failure has been associated to rhabdomyolysis in *P. falciparum* and *P.vivax* infections, probably due to the sequestration of parasitized red cells in the skeletal capillaries and consequent vessels occlusion.^108^ In the pathogenesis of ARF related to rhabdomyolysis, the myoglobin nephrotoxic effect play the principal role; hypovolemia, hypotension, fever, acidosis and the use of non-steroidal anti-inflammatory drugs may worsened the renal function.^109^,^110^

Black water fever (BWF), a rare but severe complication of severe malaria, is characterized by fever, intravascular haemolysis with haemoglobinuria, dark urines and often acute renal failure.^111^–^113^ Haemoglobin release during massive haemolysis causes renal impairment. Drugs as quinine, halophantrine and mefloquine, and G6PD deficiency, have been suggested to be the trigger of BWF.

### Placenta involvement

Malaria during pregnancy is associated to high morbidity and mortality both for the mother and the child.^114^–^117^ Mother could develop severe malaria and severe anemia, and is much exposed to obstetric adverse events.^115^,^117^–^119^ Placental malaria has been associated with elevated risk of miscarriage, preterm delivery, intrauterine growth retardation, low birth weight, fetal anemia, congenital malaria and perinatal mortality.^114^,^116^,^120^,^121^ Placental malaria is quite common during *P. falciparum* infection, less common in *P. vivax* malaria; *P. falciparum* and *P. vivax* placental mixed-infection can occur.^122^ *P. vivax* placental malaria may lead to the same adverse events as *P. falciparum* infection, even if with milder consequences.^121^–^124^

In high endemic areas, while adults are less susceptible, pregnant women are commonly susceptible to malaria infection because pregnancy causes a transient depression of cell mediated immunity. Age under 25 years and primiparity are both risk factors for developing placental malaria;^115^,^120^ moreover the risk increases in the first and second trimester of pregnancy. A meta-analysis carried out in four sub-Saharan African countries demonstrated a reduction of placental malaria in multi-gravidae women with blood group 0; however, statistically significance were demonstrated in only two of these studies.^125^

Alterations of materno-fetal blood exchange are the basis of placental malaria. During infection, parasitized red cells both from *P. falciparum* and *P. vivax* are sequestered within the placenta and they accumulate in intervillous spaces; trophozoite and schizont forms also accumulate in the placenta.^13^ The presence of IRBC activates mononuclear cells which release chemokines to recruit additional phagocytic cells in the intervillous spaces. Elevated TNF α and IL-10 levels were also described and were associated with poor pregnancy outcomes.^13^ IRBC, leukocyte infiltration, fibrin and hemozoin depositions contribute to increase the thickness of the trophoblast basement membrane and to alter the intervillous and perivillous spaces, causing reduction of oxygen and nutrient transport to the fetus.^13^,^126^

*P. falciparum* parasitized erythrocytes adhere to chondroitin sulphate A expressed in placenta; thus, placenta selects for strains of *P. falciparum* with a CSA-binding phenotype. Primigravidae who have not been exposed to *P. falciparum* CSA-binding phenotype are still susceptible to placental infection, while multigravidae have developed antibodies during the first successful pregnancies.^115^ *P. vivax* parasitized erythrocytes can also cytoadhere to the placenta but their mechanism is not clarified, yet.^5^,^124^

Molecular studies demonstrate that IRBC binding to chondroitin sulphate A (CSA), expressed on the apical membrane of the placental syncytiotrophoblast epithelium, is mediated by VAR2CSA antigen, a variant of the PfEMP1 family proteins. There are multiple genes encoding for different VAR2CSA antigens; a study performed in Cameroon shows that the parasites infecting pregnant women living in high transmission areas have an increased copy number of *var2csa* genes compared to non-pregnant women. The multiplicity of var2csa-type genes confers to *P. falciparum* parasites a greater capacity for antigenic variation and evasion of immune response.^127^ A recent study describes a new flow cytometry-based adhesion assay that use apical epithelial plasma membrane vesicles and IRBC isolated from patients.^128^ Data from this study showed that the vesicles prepared from various placental regions could adhere to IRBC in different percentage but with the same adhesion intensity; moreover, parasite molecules, other than VAR2CSA, can also mediate placental adhesion, suggesting that a different molecular pathway can occur. These findings were confirmed by another study that showed that transcripts from *var* genes, different from *var2csa*, were found in 67% of placental isolates, revealing the importance of other adhesion molecules during pregnancy.^129^

Complement activation may play an important pathogenetic role during placental malaria. C5a, a factor derived from the complement cascade, is increased in peripheral blood and placental blood of pregnant women with malaria compared with pregnant women without malaria infection.^130^ Factors derived from the activation of the complement cascade are likely to have a role in inflammation during placental malaria, in particular they could stimulate the release of pro-inflammatory cytokines and chemokines by monocytes and neutrophils.^130^,^131^ Moreover, C5a could play an important role in dysregulation of angiogenesis since it seems to stimulate monocyte production of the anti-angiogenic factor sFlt-1, a soluble variant of VEGFR-1. The sFlt-1 binds to and sequesters placental growth factor and VEGF, leading to vascular and placental alteration.^130^,^131^ Muehlenbachs et al. performed a study in Tanzanian women showing that the FLT1 genotype was associated to pregnancy loss, low birth weight, placental inflammatory gene expression and high Flt1 levels.^132^

## Conclusions

The pathogenicity of severe malaria infection is complex and it is regulated by both parasite and host factors. Cytoadherence of IRBC to the vascular endothelium and rosetting are unique features of malaria parasites which are likely to contribute substantially to the vascular damage and the consequent excessive inflammatory/immune response of the host. This can occur in many different organs, a feature that can partly explain the complexity of the clinical manifestations occurring in severe malaria. In this context, adequate clinical management of malaria patients requires first an accurate diagnosis, then appropriate antimalarial treatment, associated with adjunct supportive therapies which need to be adapted to the different clinical presentation of the disease.
